# Assessment of the effects of hyperbaric oxygen treatment on new bone formation after sinus lift in a diabetic rabbit model

**DOI:** 10.1186/s40729-025-00654-2

**Published:** 2025-10-20

**Authors:** Abdullah Tugrul Coskun, Hasan Ayberk Altug, Ömer Orkun Cevizcioglu, Aydin Özkan, Servet Güresci, Jörg Wiltfang, Aydin Gülses

**Affiliations:** 1 Department of Oral and Maxillofacial Surgery, Topraklik Oral and Dental Health Center, Ankara Provincial Health Directorate, T.R. Ministry of Health, Ankara, Turkey; 2 Department of Oral and Maxillofacial Surgery, Faculty of Dentistry, Tinaztepe University, Izmir, Turkey; 3 Department of Oral and Maxillofacial Surgery, Gulhane Faculty of Dentistry, University of Health Sciences, Ankara, Turkey; 4 Ankara Bilkent City Hospital Department of Pathology, Ankara, Turkey; 5 Department of Oral and Maxillofacial Surgery, UKSH Campus Kiel, Christian Albrecht’s University, Arnold-Heller Straße 3, Haus B, UKSH, 24105 Kiel, Germany

**Keywords:** Hyperbaric oxygen therapy, Hyperbaric oxygen treatment, Sinus lift, Alloxan-induced diabetes, BMP-2, VEGF

## Abstract

**Purpose:**

This study aims to evaluate the effects of hyperbaric oxygen treatment (HBOT) on new bone formation after sinus lift operation in diabetic rabbits.

**Methods:**

27 New Zealand White rabbits that were alloxan monohydrate-induced diabetics were divided into four groups following the sinus lift procedure. Hyperbaric oxygen treatment was applied to 14 rabbits in experimental groups (HBOT-1, HBOT-2). Subjects were sacrificed after 4 (HBOT-1, C-1) and 8 (HBOT-2, C-2) weeks. Histomorphometry and immunohistochemistry were used in histopathological evaluation.

**Results:**

The new bone area in the HBOT-1 group is statistically more significant than the C-1 group (*p* = 0.002) and the new bone area in the HBOT-2 group is statistically more significant than the C-2 group (*p* = 0.001). The area intensity and severity of staining with anti-collagen I in HBOT-1 group is statistically significant compared to C-1 group (*p* = 0.001) and in HBOT-2 group compared to C-2 group (*p* = 0.001). The area intensity and intensity of staining with anti-BMP-2 in the HBOT-1 group is statistically significant compared to the C-1 group (*p* = 0.000) and in the HBOT-2 group compared to the C-2 group (*p* = 0.001). The number of new vessels in HBOT-2 group is statistically significant compared to C-2 group (*p* = 0.000).

**Conclusions:**

Hyperbaric oxygen treatment in the long and short term increases new bone formation, and intensity the level of collagen type I, bone morphogenetic protein-2, and the vascular endothelial growth factor level, in the long term. Based on these results, it was concluded that the negative impact of diabetes on new bone formation can be recovered with hyperbaric oxygen treatment.

## Introduction

In today’s dentistry, implant applications are rapidly progressing towards becoming an excellent treatment option. For the success of implant treatments, the existing bone must be of sufficient quality and quantity. However, alveolar bone deficiencies in the area of implant application limit its use. This problem is especially common in the posterior maxilla as a result of bone resorption and sinus pneumatization. This problem, which is encountered due to insufficient bone in the posterior region of the maxilla in implant applications, can be solved with sinus lift application using various graft materials [1–8].

New bone formation in maxillary sinus augmentation depends on the rate of revascularisation and recruitment of osteoblasts. However, new bone formation in the area where the operation is performed and augmented with various graft materials can be problematic, especially due to systemic disorders present in the patient and their negative effects on osteogenesis.

Metabolic disorders characterized by diabetes and hyperglycemia adversely affect the osseointegration process of titanium implants and may lead to high rates of implant loss [9]. In prospective studies, it has been determined that the implant failure rate is high 1 year after implant application in diabetic patients [10]. For this reason, clinicians are trying to determine new treatment strategies to prevent implant loss in diabetic patients [9]. Numerous materials and methods, including lasers, ultrasonography, hyperbaric oxygen, ozone, bone grafts, growth factors, and blood products (platelet-rich plasma and platelet-rich fibrin), can be used to stimulate new bone formation and healing in order to reduce these adverse effects.

In cases involving oral, dental, and maxillofacial surgery, hyperbaric oxygen treatment (HBOT) can be included in the treatment plan in order to increase the effectiveness of the treatment and surgical procedure. Although HBOT has been used as a medical treatment for a long time, its use in the treatment of hypoxic wounds has been accepted in recent years [11]. There should be enough oxygen in the wound for the development of new cells and tissues in wound healing. Oxygen plays a critical role in collagen synthesis, matrix deposition, angiogenesis, epithelium formation, and new bone formation [12–15].

Our study, designed as a prospective, randomized, and controlled animal experiment, aimed to determine whether HBO application could reduce the negative effects of uncontrolled type I diabetes on new bone formation in the augmented area after sinus lift operation.

## Materials and methods

All procedures were approved by the institutional review board of the University of Health Sciences Gülhane Training and Research Hospital (Ankara, Turkey) with approval number 14/145. Protection of animals were clearly described and complied with national and international protection guidelines.

### Experimental model

A total of 32 male, 8–10 weeks old, New Zealand White (Oryctolagus cuniculus) rabbits with an average weight of 2.97 kg were used in the study. A diabetes model was tried to be developed in all 32 rabbits in the experimental and control groups and 5 rabbits died while waiting for their sacrification time. All of the rabbits underwent sinus lift operation and a total of 14 rabbits in the experimental group underwent HBO treatment. HBO treatment was not applied to 13 rabbits in the control group. At the end of the study, 13 of 27 rabbits were sacrificed at week 4 and 14 at week 8.

The rabbits used in the study were divided into 4 groups as group HBOT-1 (n = 7), group HBOT-2 (n = 7), group C-1 (n = 6), and group C-2 (n = 7), sacrificed at week 4 and 8 weeks, respectively.

### Development of diabetes model

Before alloxan monohydrate injection, 2 g/kg glucose was given orally to each rabbit in 10 cc distilled water. In addition, glucose was added to the drinking water of the rabbits 12 h before the alloxan injection. These treatments aimed to prevent hypoglycemia that may occur after alloxan injection.

Alloxan monohydrate at a dose of 100 mg/kg was dissolved in 10 cc saline and injected into the ear marginal veins of rabbits. Fasting blood glucose levels of the rabbits were measured with a glucose meter 7 days after alloxan administration and were found to be 200 mg/dl and above and were considered diabetic [16–18]. Lower doses of alloxan monohydrate injections were repeated in rabbits that did not develop diabetes, and 1 week later, blood glucose values were measured again. While the second dose of alloxan monohydrate rate was 75 mg/kg, the third dose was adjusted to 50 mg/kg. The total alloxan monohydrate dose administered to one rabbit did not exceed 200 mg/kg.

### Sinus lifting procedure

**Fig. 1:**
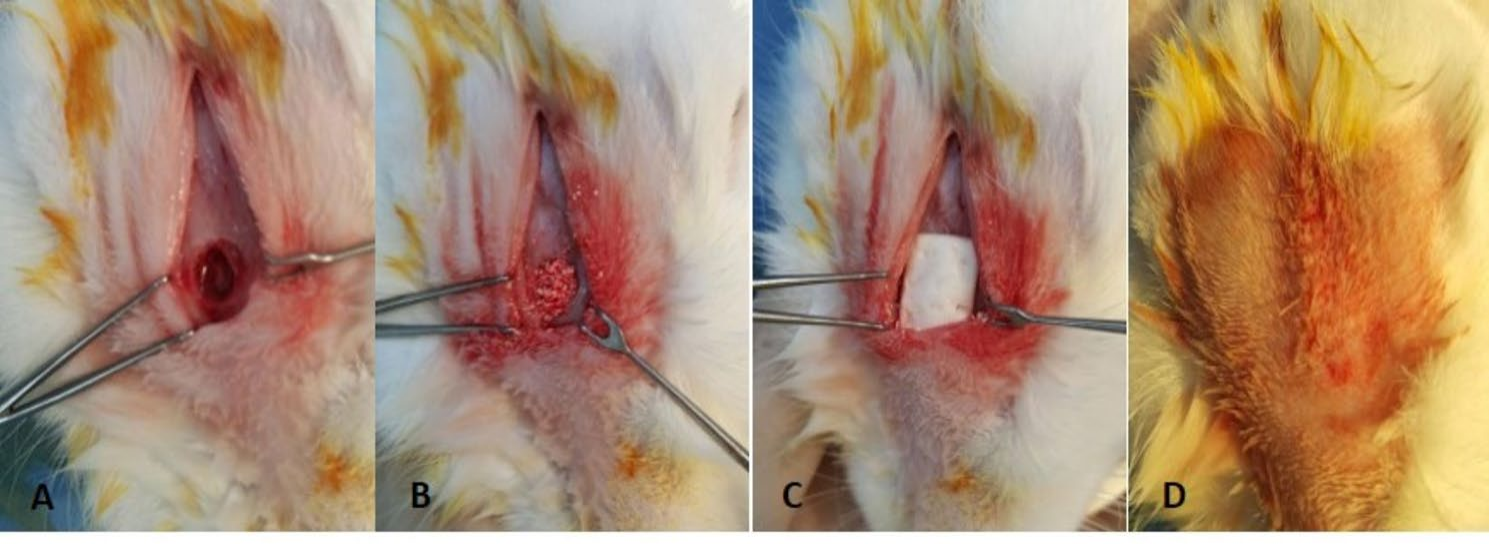
**A** Sinus membrane lifting; **B** xenograft application; **C** collagen membrane application; **D** suturing

The rabbits used in our study were anesthetized with Ketamine HCl 25 mg/kg I.M. and Xylazine HCl 5 mg/kg I.M. Artikain HCl 1 cc was injected into the midline of the nasal dorsum for local anesthesia. A 5 cm incision was made in the midline with a scalpel. Nasal bone and nasoincisal suture were exposed through the incision including the skin and periosteum. A circular bone window with a diameter of approximately 5 mm was opened with a carbide bur. This bone window was opened approximately 2 cm anterior to the nasofrontal suture and 1 cm lateral to the midline. Care was taken not to perforate the sinus membrane during this procedure. The sinus membrane was carefully elevated in an anteroventral direction. The resulting cavity was filled with 1 cc of bovine bone-derived cancellous graft material. The bone window was covered with a 10 × 10 mm collagen membrane. The periosteum and skin were closed using a resorbable 4/0 suture material (Fig. 1). The operation lasted 20–30 min and afterwards the animals were carefully monitored until fully recovered from anaesthesia. All the rabbits were administered I.M. amoxicillin (15 mg/kg) given for 3 days postoperatively.

### Hyperbaric oxygen treatment

Using 2.4 ATA (absolute atmospheres) pressure and 100% O_2_, 10 sessions of HBOT were applied to each rabbit for 90 min. In order to prevent potential discomfort and barotrauma, the pressure was increased during HBO treatment and the pressure was decreased at a low rate of 0.2 ATA per minute at the end of the treatment.

### Histopathological evaluation

At the end of the study, 27 rabbits grouped according to sacrification times were anaesthetized with ketamine HCl 35 mg/kg I.M. and xylazine HCl 5 mg/kg I.M. The rabbits were sacrificed by intracardiac administration of high-dose ketamine. The incision was made in the lip commissure of the rabbits. The maxilla was exposed through incisions made in the buccal mucosa of the upper jaw. Osteotomy was performed with a carbon disc from the nasofrontal suture and above the nose in the sinus lift area. A block excision including the palatinal part of the maxilla was performed and the necessary part for histopathological examination was removed.

The maxillectomy materials were decalcified in a 5% nitric acid solution for 5 days after tissue fixation in a 10% formaldehyde solution. Thus, the minerals in the bone tissue were removed and the tissue was made cuttable. The maxillectomy material was then sliced at 0.5 cm intervals to reveal the area where the graft material was placed. Two samples were taken from this area for each case and placed in cassettes. After 13 h of tissue tracking, the samples were embedded in paraffin blocks. Sections of 3-micron thickness were taken from the paraffin blocks and stained with hematoxylin–eosin (HE).

### Evaluation of bone morphometry

HE stained sections were subjected to the morphometric examination of new bone formation using the Nikon NIS-Elements D 3.0 program under a 4 × 0.10 objective in a Nikon i600e light microscope. Meanwhile, bone trabeculae other than the local sinus wall that developed around the graft were evaluated in terms of new bone formation areas and HE staining intensity. The newly formed bone areas around the graft material were manually determined at five different points selected within an area of 3.4 mm^2^ and an intensity of 2560 × 1920 (4,915,200) pixels (px) in each HE-stained sample and the data obtained were transferred to the Excel program. In the data obtained, bone areas were calculated in mm^2^ and HE staining intensity was calculated in pixels (px).

### Immunohistochemical method and evaluation of reactivity

For each case, 3-micron sections were taken to be used during immunohistochemical staining. After deparaffinization, which is the first step in this process, the sections were boiled in ethylenediaminetetraacetic acid (EDTA) for 20 min and then peroxidase blocking was performed using the ‘Bond Polymer Refine Detection Kit’. All cases were incubated with anti-VEGF at 1:500 dilution, anti-BMP-2 at 1:100 dilution and anti-collagen type I at 1:400 dilution for 30 min. Slides were kept in post primer for 10 min, in polymer for 10 min, in DAB-chromogen for 10 min, and in Mayer’s hematoxylin for 5 min. After dehydration in 96% alcohol for 2 times, they were kept in xylene for 2 min and covered with entellan.

During the staining process, corpus luteum for anti-VEGF, periosteum for anti-BMP-2, and dermis for anti-collagen type I were used as positive controls.

For anti-VEGF, staining of the vessel wall and/or vessel endothelium was taken as basis. The number of vessels stained with anti-VEGF in the × 10 magnification area was determined with the same brand microscope under a 40 objective.

For anti-BMP-2 and anti-collagen I, the entire extracellular matrix area within the ossification area in the section was evaluated.

### Statistical evaluation

The research data were processed and analyzed with SPSS for Windows V. 22. Descriptive statistics were given as numbers, and percentages for discrete data and mean, and standard deviation for continuous data. The chi-square test was used for the comparison of discrete variables between groups.

The difference in immunohistochemical variables between the groups was compared by one-way analysis of variance, and covariance analysis was used to evaluate the effect of hyperbaric oxygen treatment corrected for time. Bonferroni correction was used as a post hoc test. *p* < 0.05 values were considered statistically significant.

## Results

### Evaluation of new bone formation in bone morphometry

**Fig. 2:**
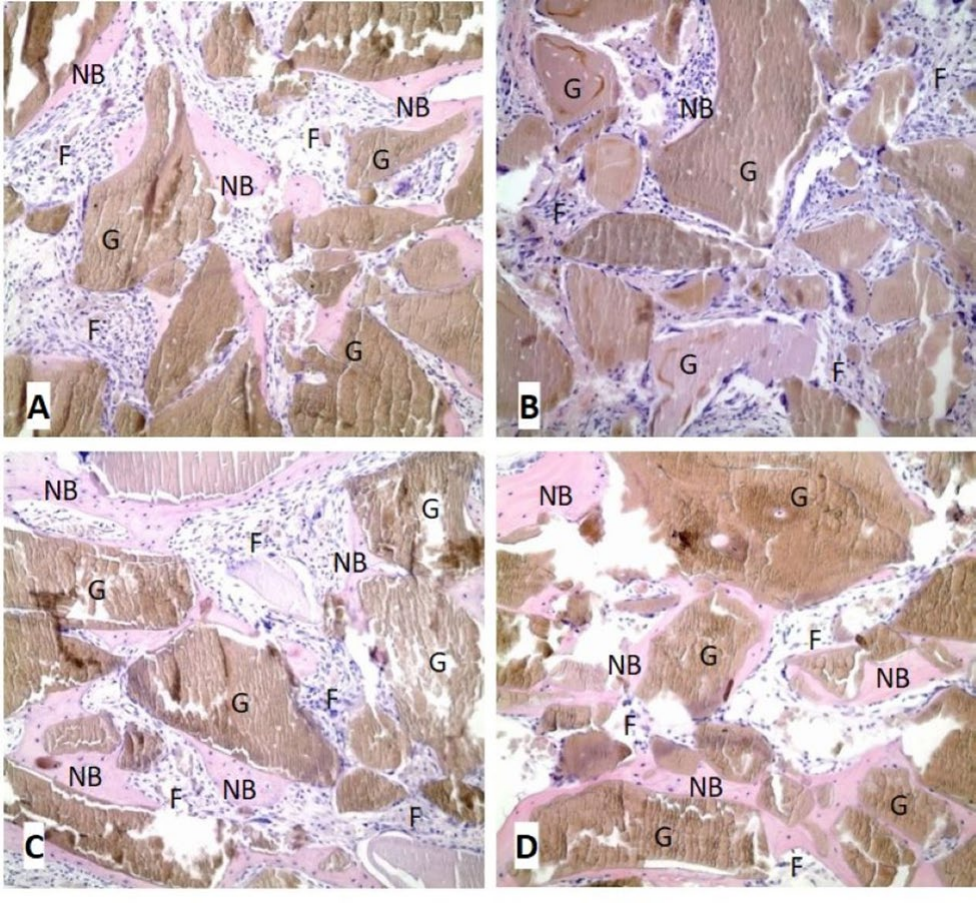
**A** group HBOT-1 (H&E, × 200); **B** group C-1 (H&E, × 200); **C** group HBOT-2 (H&E, × 200); **D** group C-2 (H&E, × 200). (G—graft; NB—new bone; F—fibroblasts and connective tissue)

As a result of histomorphometric evaluations of the subjects sacrificed in the 4th week, an average of 0.4329 mm^2^ new bone area was measured in the group HBOT-1 and 0.1182 mm^2^ in the group C-1. The HE staining intensity of the newly formed bone areas was 2,041,753 px in the group HBOT-1 and 1,126,093 px in the group C-1 (Fig. 2A, B).

In the 8th week, as a result of histomorphometric evaluations of the sacrificed subjects, an average of 0.6959 mm^2^ new bone area was measured in the group HBOT-2 and 0.2453 mm^2^ in the group C-2. When the HE staining intensity of the newly formed bone regions is examined, it is 4,619,980 px in the group HBOT-2 and 2,510,093 px in the group C-2 (Fig. 2C, D).

### Evaluation of immunoreactivity in new bone formation

**Fig. 3:**
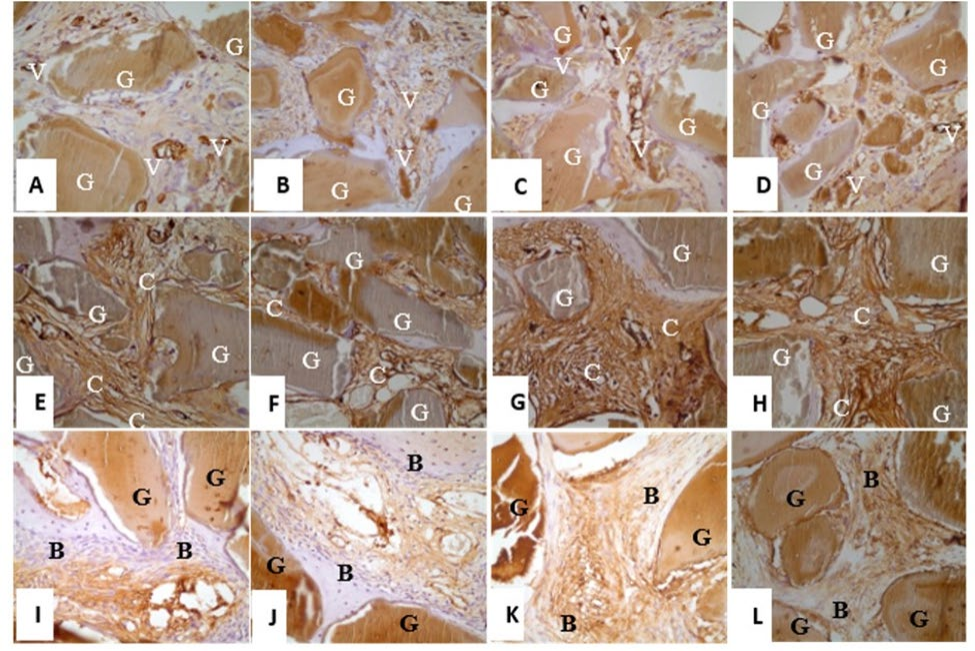
**A** group HBOT-1 (anti VEGF × 400); **B** group C-1 (anti VEGF × 400); **C** group HBOT-2 (anti VEGF × 400); **D** group C-2 (anti VEGF × 400); **E** group HBOT-1 (anti-Coll A1 × 400); **F** group C-1 (anti-Coll A1 × 400); **G** group HBOT-2 (anti-Coll A1 × 400); **H** group C-2 (anti-Coll A1 × 400); **I** group HBOT-1 (anti-BMP-2 × 400); **J** group C-1 (anti-BMP-2 × 400); **K** group HBOT-2 (anti-BMP-2 × 400); **L** group C-2 (anti-BMP-2 × 400). (G—graft; V—VEGF in connective tissue; C—collagen; B—BMP-2 in connective tissue)

In the 4th week, the number of vessels stained with anti-VEGF as a result of immunohistochemical evaluation of the sacrificed subjects was found to be 58.14 in the group HBOT-1 and 71 in the group C-1 (Fig. 3A, B).

In the 8th week, the number of vessels stained with anti-VEGF as a result of immunohistochemical evaluation of the sacrificed subjects was found to be 87.14 in the group HBOT-2 and 42.86 in the group C-2 (Fig. 3C, D).

In the 4th week, the percentage of the area stained with anti-collagen type I as a result of immunohistochemical evaluation of the sacrificed subjects was 28.57% in the group HBOT-1 and 16.67% in the group C-1 (Fig. 3E, F).

In the 8th week, the percentage of the area stained with anti-collagen type I as a result of immunohistochemical evaluation of the sacrificed subjects was 88.57% in the group HBOT-2 and 28.57% in the group C-2 (Fig. 3G, H).

In the 4th week, the percentage of area stained with anti-BMP-2 as a result of immunohistochemical evaluation of the sacrificed subjects was 65% in the group HBOT-1 and 47.50% in the group C-1 (Fig. 3I, J).

In the 8th week, the percentage of the area stained with anti-BMP-2 as a result of immunohistochemical evaluation of the sacrificed subjects was 75.71% in the group HBOT-2 and 52.86% in the group C-2 (Fig. 3K, L).

### Statistical evaluation of new bone formation

According to the Post Hoc test performed between the HBOT and control groups sacrificed at 4 and 8 weeks, when the area and density of new bone formation were compared, a statistically significant difference was observed in the group HBOT-2 compared to the group C-2 (*p* = 0.001). In the group HBOT-1, a statistically significant difference was found when the new bone area was compared with the group C-1 (*p* = 0.002), but no significant difference was found in bone density (*p* = 0.374) (Table 1).

**Table 1:** Multiple comparison results of newly formed bone areas and densities between groups

Dependent variable	Group (I)	Group (J)	Average difference (I − J)	Std. error	Difference (P)
*Multiple comparison*					
Area (mm^2^)	HBOT-2	HBOT-1	0.26303	0.07073	0.007
		K-2	0.45066	0.07073	0.001
		K-1	0.57776	0.07361	0.000
	HBOT-1	HBOT-2	− 0.26303	0.07073	0.007
		K-2	0.18763	0.07073	0.085
		K-1	0.31473	0.07361	0.002
	K-2	HBOT-2	− 0.45066	0.07073	0.001
		HBOT-1	− 0.18763	0.07073	0.085
		K-1	0.12710	0.07361	0.586
	K-1	HBOT-2	− 0.57776	0.07361	0.000
		HBOT-1	− 0.31473	0.07361	0.002
		K-2	− 0.12710	0.07361	0.586
Density (px)	HBOT-2	HBOT-1	2,578,227	448,960.5	0.000
		K-2	2,109,887	448,960.5	0.001
		K-1	3,493,887	467,292.9	0.000
	HBOT-1	HBOT-2	− 2.6E+6	448,960.5	0.000
		K-2	− 468,339	448,960.5	1.000
		K-1	915,660.2	467,292.9	0.374
	K-2	HBOT-2	− 2.1E+6	448,960.5	0.001
		HBOT-1	468,339.5	448,960.5	1.000
		K-1	1,384,000	467,292.9	0.042
	K-1	HBOT-2	− 3.5E+6	467,292.9	0.000
		HBOT-1	− 915,660	467,292.9	0.374
		K-2	− 1.4E+6	467,292.9	0.042

When the number of vessels stained with anti-VEGF was compared between the groups, no significant difference was found between HBOT and control groups sacrificed at weeks 8 and 4. However, a significant difference was observed between group HBOT-2 and group C-1 (*p* = 0.001) (Table 2).

**Table 2:** Multiple comparison results of the number of vessels stained with anti-VEGF between groups

Dependent variable	Group (I)	group (J)	Average difference (I − J)	Std. error	Difference (P)
*Multiple comparison*					
VEGF	HBOT-2	HBOT-1	29.000	3.334	0.000
		K-2	44.286	3.334	0.000
		K-1	16.143	3.470	0.001
	HBOT-1	HBOT-2	− 29.000	3.334	0.000
		K-2	15.286	3.334	0.001
		K-1	− 12.857	3.470	0.007
	K-2	HBOT-2	− 44.286	3.334	0.000
		HBOT-1	− 15.286	3.334	0.001
		K-1	− 28.143	3.470	0.000
	K-1	HBOT-2	− 16.143	3.470	0.001
		HBOT-1	12.857	3.470	0.007
		K-2	28.143	3.470	0.000

When the percentage of area stained with anti-collagen type I was compared, a statistically significant difference was found between HBOT and control groups sacrificed at both the 4th and 8th weeks (*p* = 0.001) (Table 3).

**Table 3:** Multiple comparison results of the percentage of area stained with anti-collagen type I between groups

Dependent Variable	Group (I)	Group (J)	Average difference (I − J)	Std. error	Difference (P)
*Multiple comparison*					
Collagen type I	HBOT-2	HBOT-1	60.000	2.468	0.000
		K-2	60.000	2.468	0.001
		K-1	71.905	2.569	0.000
	HBOT-1	HBOT-2	− 60.000	2.468	0.000
		K-2	0.000	2.468	1.000
		K-1	11.905	2.569	0.001
	K-2	HBOT-2	− 60.000	2.468	0.001
		HBOT-1	0.000	2.468	1.000
		K-1	11.905	2.569	0.001
	K-1	HBOT-2	− 71.905	2.569	0.000
		HBOT-1	− 11.905	2.569	0.001
		K-2	− 11.905	2.569	0.001

In the statistical evaluation of the percentage of area stained with anti-BMP-2, a significant difference was observed both in the subjects sacrificed at week 8 (HBOT-2, C-2) (*p* = 0.001), and at week 4 (HBOT-1, C-1) (*p* = 0.000) (Table 4).

**Table 4:** Multiple comparison results of the percentage of area stained with anti-BMP-2 between groups

Dependent variable	Group (I)	Group (J)	Average difference (I − J)	Std. error	Difference (P)
*Multiple comparison*					
BMP-2	HBOT-2	HBOT-1	10.714	3.056	0.011
		K-2	22.857	3.056	0.001
		K-1	28.214	3.181	0.000
	HBOT-1	HBOT-2	− 10.714	3.056	0.011
		K-2	15.286	3.056	0.004
		K-1	− 12.857	3.181	0.000
	K-2	HBOT-2	− 22.857	3.056	0.001
		HBOT-1	− 15.286	3.056	0.004
		K-1	− 28.143	3.181	0.634
	K-1	HBOT-2	− 28.214	3.181	0.000
		HBOT-1	− 17.500	3.181	0.000
		K-2	− 5.357	3.181	0.634

## Discussion

In our study, alloxan monohydrate was used to create an experimental diabetes model because of its selective cytotoxic effect on β cells of pancreatic islets of Langerhans. Streptozotocin and alloxan monohydrate are the two most commonly used preparations to develop a chemical model of diabetes in rabbits. However, alloxan monohydrate has several superiorities and advantages, especially in the development of a long-term type I diabetes model. A few months after the administration of a diabetic agent, blood glucose may start to decrease as a result of the regeneration of β cells or the development of islet cell adenoma. This oncogenic effect (adenoma risk) is higher in streptozotocin diabetes compared to alloxan. Therefore, alloxan diabetes is more advantageous when it is necessary to establish long-term diabetes and monitor chronic complications. Wang et al. [17] reported that rabbits and alloxan monohydrate can be used safely to develop a diabetes model lasting longer than 1 year. Although studies have indicated that the maximum diabetic dose of alloxan is 40–45 mg/kg I.V., it is used in a wide dose range. According to the literature, alloxan monohydrate is frequently used in the dose range of 80–150 mg/kg [16]. In their study, Younis et al. [18] administered a single dose of 120 mg/kg alloxan monohydrate via ear vein to rabbits in diabetic groups, while Vieira et al. [16] administered a single dose of 90 mg/kg alloxan monohydrate. In our study, the dose of alloxan monohydrate used to develop an experimental diabetes model was 100 mg/kg, and rabbits with a blood glucose level equal to or greater than 200 mg/dl after 1 week were considered diabetic.

Insulin is the main hormone that regulates glucose metabolism and shows anabolic effect on bone. In a study conducted in diabetic rats, it was found that general or local insulin administration positively affected the connection at the implant-bone interface [9]. In parallel with this result, Malekzadeh et al. [19] showed that insulin can eliminate the negative effects of diabetes on bone healing and increase bone formation around the implant. Since the positive effect of insulin on bone formation in diabetic organisms has been shown by studies, systemic or local insulin administration was not performed in order to provide and maintain an uncontrolled type I diabetes model in a total of 27 rabbits belonging to the experimental and control groups used in our study.

In our study, the technique of entering the sinus cavity through the nasal dorsum of rabbits was used as a sinus lift model. This technique was described by Xu et al. [20]. In addition, Watanabe et al. [7] used the intraoral approach as a sinus lift model in rabbits. In our study, we preferred to use the extraoral approach described by Xu et al. [20] because wound healing may be adversely affected by diabetes, weight loss is expected due to the effect of diabetes, and oral feeding of rabbits should not be adversely affected. According to Asai et al. [21], the sinus lift model performed through the nasal ostium in the rabbit is considered to be a good model for understanding the pathological and physiological atrophy mechanism in the sinus floor.

A total of 14 rabbits in the experimental group of our study were subjected to HBO treatment with 100% O_2_ at a pressure of 2.4 ATA for 90 min, 10 sessions, and once a day. In the literature, HBO treatment protocol differs in animal and in vitro studies. However, the use of 100% O_2_ at a pressure range of 1.5–3.0 ATA and 60–90 min is more common. According to Suer et al. [15], HBO treatment applied once a day accelerates bone repair and new vessel formation more than twice a day.

It is important that the sessions are performed at the same time of the day when applying HBO treatment. Helmers et al. [22] conducted an experimental study to show that HBO treatment stimulates vascular regeneration in wound healing. Accordingly, they removed a half-thickness flap in the palatinal mucosa in 10 New Zealand White rabbits. They divided the rabbits into two groups and applied HBO treatment to 5 rabbits in the experimental group for a total of 10 sessions at 2.5 ATA pressure and 90 min. As a result of the study in which they compared the HBOT and control groups, they stated that HBO treatment increased the vascular regeneration capacity in the wound, especially on the 7th, 9th, and 11th days in the healing of keratinized oral mucosal flap. In our study, in parallel with the study of Helmers et al. [22], we planned and applied HBO treatment in 10 sessions, once a day and at the same time every day.

Diabetic wounds, peripheral neuropathy, vascular diseases, and local immunity are also associated with the problem. With impaired inflammation and healing, chemotaxis, phagocytosis, bacterial death, and lymphocytic functions are reduced. Morbidity and mortality rates are high. HBO treatment has been used in diabetic foot ulcers for more than 50 years [23, 24]. There are many experimental studies on the treatment of diabetic complications with HBO. Regarding this issue, Oliveira et al. [10] suggested that HBO treatment improves osteointegration by reducing the negative effects of diabetes in diabetic rats. In their study, they applied HBO at a pressure of 2.0 ATA to each rat in the experimental group. As a result of the study, they found that HBO treatment compensated for the negative effect of diabetes on bone healing around the implant when applied before or after the surgical procedure. On the other hand, they claimed that HBO treatment after implant application to healthy rats decreased bone-implant connection (BIC). It was also reported in this study that HBO treatment did not affect blood glucose levels.

Tuk et al. [25] examined the effect of HBO treatment on wound healing in diabetic rats and applied 100% oxygen under 2.4 ATA pressure for 90 min to the rats in the experimental group. In this study, they stated that they determined the positive effect of HBO treatment on diabetic wound healing. In the same study, 15% weight loss was detected in rats for 3.5–4 weeks with diabetes induction. In this study, we also detected weight loss while developing a diabetes model in rabbits. In addition, in parallel with the application of Tuk et al. [25], we found it appropriate to perform a total of 10 sessions of 90 min of HBO treatment at a pressure of 2.4 ATA.

In experimental animal models, HBO treatment has been shown to stimulate angiogenesis in wound tissue by increasing blood flow [23]. In addition, HBO treatment increases osteogenesis by activating osteoblasts and makes the immune system more effective by increasing phagocytic capacity.

Grassmann et al. [26] investigated the effects of HBOT in critical diaphyseal defects in their experimental study. For this purpose, they created a 15 mm diameter critical defect in the diaphysis of the right radial bone in New Zealand White rabbits. They applied an autogenous bone graft from iliac bone to the defect area and claimed that HBO treatment increased angiogenesis and new bone formation in the region. In addition, they stated that HBO increased bone healing in critical bone defects and increased the absorption of autogenous bone graft. In our study, we used bovine cancellous graft material instead of autogenous bone as graft material in sinus lift operation. In this way, in parallel with this study, we prevented the absorption of graft material by HBOT.

Suer et al. [15] investigated the effect of HBO treatment on new bone formation during periosteal distraction in rabbits. In their study, 24 adult male New Zealand White rabbits were used, and a handmade distractor device was applied to the lateral surface of the mandible corpus. They applied HBO treatment to 12 rabbits. A similar distraction protocol was applied to both groups 7 days after the latent period. HBO treatment was planned at a pressure of 2.4 ATA and a total of 20 sessions. Both groups were divided into two subgroups and sacrificed at 4 and 8 weeks. New bone formation was evaluated by photodensitometric and histological analysis. According to the results of this study, there was no statistically significant difference in new bone formation between the group HBO sacrificed during the consolidation period at week 4 and the normobaric oxygen group sacrificed during the consolidation period at week 8. However, better bone formation was found in the group HBO sacrificed at week 8 compared to the control group sacrificed at week 8. According to the researchers, periosteal distraction and HBO application can be used together to increase the quality and quantity of bone in new bone formation. Similar to the results of this study, we found a statistically significant increase in new bone area and density in the group HBOT-2 sacrificed at week 8 compared to the group C-2 (*p* = 0.001).

Younis et al. [18] immunohistochemically evaluated the changes in the amounts of growth factors in insulin-treated and non-insulin-treated diabetic rabbits. In this study, 48 New Zealand White rabbits were divided into 3 groups as control, diabetic, and insulin-treated diabetic groups. They administered a single dose of 120 mg/kg alloxan monohydrate through the ear vein to the rabbits in the diabetic groups, and the rabbits with a blood glucose level equal to or higher than 200 mg/dl after 1 week were accepted as diabetic. They extracted the lower incisors of all rabbits used in the study and sacrificed the animals on days 2, 10, 20, and 30. They immunohistochemically evaluated the levels of VEGF, TGF β-3, IGF-1R, and BMP-4 in the samples obtained. TGF β-3 is used in the evaluation of osteoblasts, fibroblasts, and osteocytes in the early healing period (days 2–10). Statistically significant differences were found in the level of TGF β-3 between the control and insulin-untreated diabetic groups. When the VEGF level was evaluated, no significant difference was found between the control group and the insulin-treated diabetic group. IGF-1R level was released more in the untreated diabetic group compared to the other groups. BMP-4 showed a statistically significant difference between the non-insulin-treated diabetic group and the insulin-treated diabetic group.

The current article has two major limitations that should be addressed to better align the experimental model with clinical relevance. First, the follow-up period employed in the study is notably shorter than the clinically recommended healing phase following a sinus lift procedure. Clinical protocols consistently suggest a healing period of at least 6 months to allow sufficient time for osseointegration and graft consolidation before implant placement. This 6-month duration is widely accepted in the literature and reflected in postoperative guidelines for sinus augmentation procedures [27]. Second, significant anatomical discrepancies exist between human and rodent maxillary sinuses, which limit the translational applicability of the findings. Unlike rodents, edentulous human maxillae frequently exhibit alveolar bone atrophy and various anatomical variations—such as the presence of maxillary sinus septa or multipartite sinus cavities—that can considerably influence the surgical outcome. These features are generally absent or insufficiently modeled in rodent subjects. For example, a meta-analysis of cone-beam computed tomography (CBCT) studies covering 12,536 human sinuses reported that maxillary sinus septa were present in 34.7% of cases [28]. Similarly, a broader systematic review of 62 studies comprising 14,664 sinuses found a 33.2% prevalence rate [29]. These data underscore that approximately one-third of human maxillary sinuses exhibit septa—an anatomical variation not adequately represented in rodent models.

## Conclusions

Consequently, hyperbaric oxygen treatment in the long and short term increases new bone formation, and intensity the level of collagen type I, bone morphogenetic protein-2, and the vascular endothelial growth factor level, in the long term. Based on these results, it was concluded that the negative impact of diabetes on new bone formation can be recovered with hyperbaric oxygen treatment.

## Data Availability

No datasets were generated or analysed during the current study.

## Abbreviations

HBOT: Hyperbaric oxygen treatment
C: Control
ATA: Absolute atmospheres
HE: Hematoxylin–eosin
VEGF: Vascular endothelial growth factor
BMP-2: Bone morphogenetic protein-2
px: Pixels
EDTA: Ethylenediaminetetraacetic acid
TGF β-3: Transforming growth factor-β
IGF-1R: Insulin-like growth factor receptor
